# Effect of Hot Water Bottle and Cloth Blanket on Rectal Temperature During Magnetic Resonance Imaging of the Head in Cats Under General Anesthesia

**DOI:** 10.3390/life14121646

**Published:** 2024-12-11

**Authors:** Ruxandra Pavel, Cristina Fernoagă, Alexandru Gabriel Neagu, Ruxandra Costea

**Affiliations:** Faculty of Veterinary Medicine, University of Agronomic Sciences and Veterinary Medicine of Bucharest, 011464 Bucharest, Romania; cristina.fernoaga@fmvb.usamv.ro (C.F.); ruxandra.costea@fmvb.usamv.ro (R.C.)

**Keywords:** cat, body temperature, anesthesia, diagnostic imaging, hypothermia

## Abstract

Maintaining an animal’s body temperature during magnetic resonance imaging (MRI) poses great challenges, as many temperature measuring devices and warming systems are incompatible with the MRI machine. The aim of this study was to examine body temperature changes and evaluate the impact of using a hot water bottle and a cloth blanket on rectal temperature during magnetic resonance imaging in cats. We included in this study 30 cats from different breeds that underwent magnetic resonance imaging for 60 min that were randomly divided into a passively insulated group (G1) covered with a blanket (*n* = 15) and a positively heated group (G2) using a silicone hot water bottle under the abdomen and the same cloth blanket over the cat (*n* = 15). The body temperature was measured before premedication, before induction of anesthesia, and after the MRI examination. Body temperature decreased slightly but significantly (*p* < 0.05) after premedication. At the end of the MRI, body temperature had decreased more in G1 than G2 (*p* = 0.033) to 37.0 (36.5–37.5) °C and 38 (37.9–38.2) °C, respectively. This study provides clinical evidence that cats needing magnetic resonance imaging of the head can be protected from hypothermia by using a hot water bottle placed underneath their abdomen and a cloth blanket covering their full body.

## 1. Introduction

The use of MRI to diagnose neurological disorders in cats is on the rise [1]; however, the cold examination environment and the restriction on commercial active heating devices during the procedure contribute to the risk of hypothermia, as seen in dogs and rats [2,3,4]. For a successful MRI in humans, the patient must remain motionless to achieve high-quality images [5], which means animals require at least deep sedation or general anesthesia [6]. General anesthesia greatly impacts thermoregulation [7], often leading to hypothermia, which is the most common complication in small animal patients. Studies show that up to 84% [8,9] of these patients experience hypothermia, with the incidence reaching 64% during thoracolumbar surgery [10]. Moreover, the side-effects of anesthesia can aggravate conditions in cats with neurological disorders [1,11]. Hypothermia can result in serious, life-threatening complications, such as a higher risk of delayed recovery, wound infections (mainly observed with more pronounced hypothermia, below 35 °C, and not documented in domestic animals), coagulation disorders, and bradyarrhythmia [12,13]. Maintaining body temperature during anesthesia in patients undergoing MRI is challenging. MRI scanners rely on magnetic fields and radio waves to generate body images, requiring all electrical devices used in the scanner to be non-magnetic and electrically non-conductive. Additionally, MRI rooms are kept at low temperatures to ensure the magnet remains cool [14]. MRI rooms are typically kept at around 20 °C to operate the equipment, which can be quite cold for anesthetized animals.

A previous study demonstrated that using an insulation device made from bubble wrap and down cloth blankets could reduce the decrease in body temperature of the patients during MRI examinations that last between 30 min and 1 h. However, even with insulation, a considerable decrease in body temperature was noted during MRI examinations that lasted 2 h [15].

In this study, we evaluated a method to prevent hypothermia in cats during MRI examinations. We compared the use of a hot water bottle placed under the abdomen along with a cloth blanket to using a cloth blanket alone. Our hypothesis was that using a silicone hot water bottle together with a cloth blanket would prevent hypothermia in cats undergoing an MRI of the head.

## 2. Materials and Methods

The owners provided consent for their animals to be included in this study. This study was conducted on 30 feline patients, with a mean age of 4.5 years old, belonging to different breeds. In this study, cats meeting the following criteria were included: cats that needed an MRI examination of the head; those with a rectal temperature before premedication of 38 °C–39.2 °C; those with an American Society of Anesthesiologists (ASA) physical status of 2 and 3 [16], and those that had a complete MRI examination within 1 h. No animals were excluded from this study, as all met the inclusion criteria. Participants were randomly assigned to either the control group (G1) or the heat group (G2) using a computer-generated randomization sequence to minimize selection bias (www.random.org, accessed on 1 October 2024). A comprehensive and calm preanesthetic physical examination was conducted, assessing cardiovascular (electrocardiogram and pulse oximetry) and respiratory functions (counting breaths per minute by observing chest movements). Body temperature was recorded 15 min after the cats arrived at the clinic using a digital thermometer, and hydration levels were assessed through clinical signs such as skin turgor and mucous membrane moisture. We evaluated the color and moisture of mucous membranes (e.g., gingiva) to assess oxygenation and hydration. Blood samples were taken to further assess the cat’s overall health, in addition to the neurological examination. We assessed baseline physiological parameters to ensure that the biochemical and hematological values would not confound the results. Blood samples were used to check for markers of systemic inflammation or metabolic imbalances, ensuring that all included cats were stable and comparable as we took into account the patients that were within the established reference ranges provided by the biochemical (BA400, BioSystems, Barcelona, Spain) and hematological (Advia 2120i, Siemens Medical, Swords, Irland) analyzer. All patients suspected to have increased intracranial pressure due to brain tumors were under specific treatment closely managed by our neurologist, treatment that included diuretics, corticosteroids, or anticonvulsants. No animals were excluded from this study, as all met the inclusion criteria.

Preparation of the animals included 6 h of fasting with free access to water before the MRI. Medication and protocols were designed to minimize risk and provide consistent conditions for comparing temperature regulation methods.

All cats were premedicated with 3 mcg/kg of dexmedetomidine (Dexdomitor^®^ Zoetis, Orion pharma 0.5 mg/mL, Espoo, Finland) and 0.3 mg/kg of butorphanol (Butomidor^®^ 10 mg/mL, VetViva Richter GmbH, Wels, Austria) intramuscularly (IM). The premedication protocol provided effective sedation and analgesia, minimizing patient stress and promoting a stable recovery. After premedication, a 24 G catheter was placed in the cephalic vein of all animals. Induction of anesthesia was started with a 2 mg/kg loading dose of propofol (Fresofol^®^ 1% MCT/LCT, Fresenius Kabi Australia, Mount Kuring-gai, Australia) intravenously (IV) given in 15 s, followed by laryngeal desensitization with 0.5 mg/kg of 2% lidocaine (Xilina 2%, 2 mL, S.C. Zentiva SA, Romania, Bucharest) withdrawn with a 1 mL syringe and applied on the larynx.

We waited an additional 15 s before attempting to intubate the cats. If the patient showed resistance, we administered another 1 mg/kg bolus of propofol.

Isoflurane (Isothesia, 1000 mg/g, Piramal Critical Care B.V., Voorschoten, The Netherlands) in oxygen (500 mL + 10 mL/kg) was used to maintain anesthesia at a vaporizer setting of 2% through an anesthesia machine (AEON 7200A, Beijing, China) with the use of a rebreathing system.

End-tidal CO_2_ (EtCO_2_) was measured with a sidestream capnograph, and all cats kept their EtCO_2_ between 35 and 45 mmHg. Blood pressure was monitored noninvasively using an oscillometric method. A 2.5 cm cuff was positioned at the base of the tail to ensure accurate measurements. The monitoring equipment (Comen, STAR8000-V, Shenzhen, China) was placed outside the MRI machine to avoid interference with the imaging system. During the MRI procedure, the mean arterial pressure (MAP) was consistently maintained within normal limits (MAP > 60 mmHg). We applied an ocular lubricant (Claromed^®^, 30 g, 1% hyaluronic acid, Inmed, Bucharest, Romania) to all cats to protect the cornea during the MRI examination.

**Figure 1:**
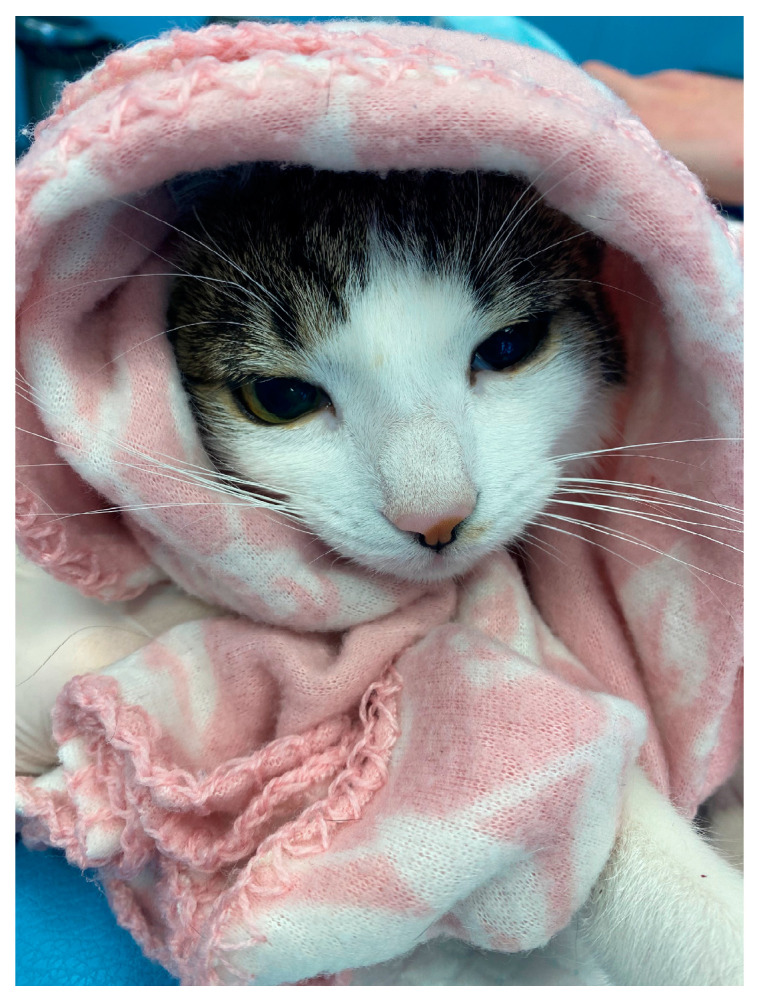
Patient from G1 in the recovery time after the MRI examination.

The cats were randomly allocated into two groups using a computer-generated randomization sequence to reduce selection bias. The first group (G1), designated as the control group (Figure 1), was covered with a 100% polyester cloth blanket measuring 160 cm × 120 cm. The second group (G2, heat group) had a silicone hot water bottle (35 cm × 20 cm) placed under their abdomen, with their entire body also covered by the same polyester cloth blanket. The control group (G1, n = 15) had a median age of 5 (6–2) years old, belonging to different breeds represented by British Shorthair (n = 4), Bengal (n = 1), Domestic Shorthair (n = 4), Ragdoll (n = 1), Main Coon (n = 1), Scottish Fold (n = 2), and Siamese (n = 2). The positively heated group (G2, n = 15) had a median age of 5 (7–3) years old, belonging to different breeds represented by Main Coon (n = 1), British Shorthair (n = 3), Domestic Shorthair (n = 6), Angora (n = 1), Exotic Shorthair (n = 1), Persian (n = 1), Birman (n = 1), and Siamese (n = 1).

**Figure 2:**
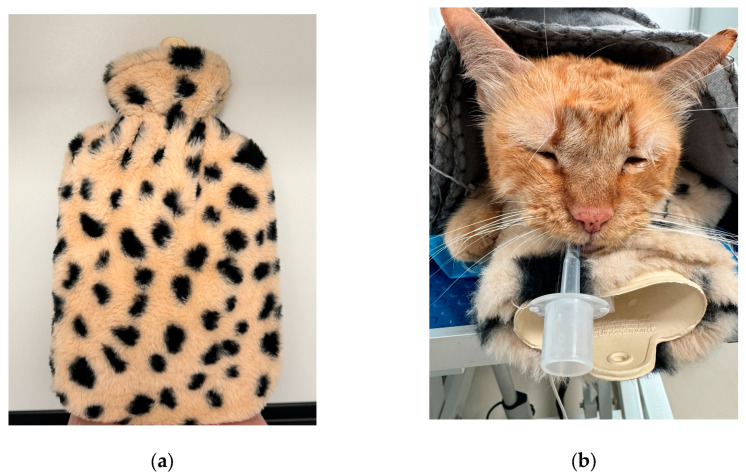
(**a**) The hot water bottle used for this study; (**b**) position of the hot water bottle along with the cloth blanket.

The hot water bottle was made entirely of silicone and was covered with a soft fleece to prevent burning the patient (Figure 2a). It was placed underneath the abdomen of each cat, and a cloth blanket was placed over the body as for G1 (Figure 2b).

The temperature of the water inside the bottle at the beginning of the MRI was 60 °C and that at the end was 55 (54–56) °C. The temperature was measured using a calibrated electronic kitchen thermometer (Westmark, Sauerland Region, Elspe, Germany). The volume of the water added to the bottle was 1.5 L.

The body temperature of the animals was measured rectally using a digital thermometer (Laica S.p.A., Viale del Lavoro, Italy) (which was calibrated at the beginning of each day of measurements) before premedication (15 min after they arrived in the clinic) at the time of anesthesia induction (before giving the propofol—10 min after the premedication) and at the end of the MRI examination (after 1 h). Calibration of the thermometer was represented by adding ice-cold water into a bowl, letting it rest for 2 min, and then inserting the tip of the thermometer on the surface. We took two measurements that showed 0 °C, and then we adjusted the thermometer by pressing and holding the reset button. The flexible tip of the thermometer was inserted about 2.5 cm into the patient’s rectum.

Efforts were made to maintain full contact between the thermometer and the rectal mucosa to reduce any interference from fecal matter during measurement. The measurement started once the thermometer was correctly positioned in the rectum and concluded when an acoustic signal indicated completion. The temperature inside the MRI room was maintained at 20 °C. The cats were placed in the prone position with their head inside the coil. All MRI scans were acquired using high-field-strength 1.5-Tesla magnets (Magnetom Essenza, Siemens Healthineers, Shenzhen, China) with a 16-channel head and neck coil. For this study, a complete MRI series was defined as including, at minimum, sagittal, transverse, and dorsal T2-weighted images, transverse fluid-attenuated inversion recovery (FLAIR), and transverse pre-contrast and post-contrast T1-weighted images. All measurements were conducted around the same time of the day, between 4 p.m. and 7 p.m., to account for potential variations in the temperature around the day. Additionally, the temperature measurements were performed by the same individual.

All cats underwent the same duration of anesthesia, which was 1 h. Patients were closely monitored during the recovery period. Recovery time was defined as the interval from extubation to the moment the cat was able to stand unassisted. Observations were continuous, ensuring an accurate assessment of each animal’s recovery progress. A statistical analysis of the data was performed on the web application DATAtab (DATAtab Team 2024. DATAtab: Online Statistics Calculator. DATAtab e.U. Graz, Austria. https://datatab.net, accessed on 12 October 2024) and Microsoft Excel (Version 16.82, 2024).

Median values, along with the range, were calculated for the temperature results. A Mann–Whitney U nonparametric statistical test was performed for the evaluation of body temperature changes in the control and heat groups, along with the differences between groups regarding duration of MRI scanning, body weight, ASA classification, anesthesia duration, and recovery time.

For more than two nominal variables, the comparison was conducted through the Kruskal–Wallis test. Statistical significance was defined as a *p*-value <0.05.

## 3. Results

There were no significant differences in body weight, time under anesthesia, or ASA classification between the two groups. The MRI diagnosis of the patients from G1 was represented by idiopathic epilepsy (5), glioma (3), meningioma (2), internal bilateral otitis (1), inflammatory process in the right bulla (1), right hemisphere tumor (1), left hemisphere posttraumatic lesion (1), and encephalitis (1). For G2, the MRI diagnosis was meningioma (4), idiopathic epilepsy (3), encephalitis (2), meningoencephalitis (2), glioma (2), interstitial edema (1), and bilateral frontal ophthalmic cyst (1).

The body temperature before premedication was 38.9 (38.7–39.0) °C in both groups (*p* = 0.099). The body temperatures before induction of anesthesia for G1 and G2 were 38.4 (37.8–38.7) °C and 38.4 (38.1–38.7) °C, with a *p*-value of 0.41. Following the MRI, the temperatures were 37.0 (36.5–37.5) °C and 38 (37.9–38.2) °C in G1 and G2, respectively, and only the value in G1 was significantly different from the value prior to the MRI (*p* = 0.033).

The recovery times for G1 and G2 were 17 (25–13) minutes and 11 (15–7) minutes, respectively, which were statistically significantly different (*p* < 0.001).

Besides the medications administered and ASA classification, the length of anesthesia is also an important factor contributing to hypothermia during anesthesia.

## 4. Discussion

Based on the findings of this study, the results suggested that putting a hot water bottle underneath the patient’s abdomen, along with a cloth blanket that covers the entire patient, prevented any temperature decrease for the allotted period of MRI examination of the head in cats.

Hypothermia can delay recovery through multiple mechanisms. In our study, the control group exhibited a longer recovery time (measured as time to standing) than cats in the heat group. This is likely due to hypothermia’s effect on reducing hepatic blood flow and enzyme activity, which slows the metabolism of anesthetic drugs. We expected that minimizing hypothermia—using a hot water bottle under the abdomen and covering the cats’ bodies—would enhance recovery time and help prevent other hypothermia-related complications [17]. Patients with lower body temperatures may take longer to recover from anesthesia, as they need to eliminate a larger amount of inhalant anesthetic. However, while the solubility of inhalant agents like isoflurane and enflurane does increase with decreasing temperature, the effect is relatively small—approximately 6% per degree of temperature drop [18]. Thus, based on the temperature range observed in this study, this factor is unlikely to have a significant effect on anesthetic clearance during recovery.

The initial decline in body temperature during anesthesia is largely attributed to shifts in blood distribution triggered by anesthetic agents, as seen in humans [19], but we do not have any data regarding animals.

Small animals lose body heat faster than larger ones because they have a higher surface-area-to-mass ratio [20], which accelerates heat loss through convection, conduction, radiation, and evaporation. In this study, the drop in rectal temperature observed can be explained by several factors.

The use of a cloth blanket likely reduced heat loss through convection and conduction by insulating the animals and limiting heat transfer to the surrounding air and surfaces. Additionally, placing a hot water bottle under the abdomen warmed the air around the animal, which helped decrease heat loss through radiation by creating a localized warm zone. Decreased muscle activity due to anesthesia, along with the drugs’ effects on the hypothalamus [21] and central alpha-2 adrenoceptors [22], also contributed to reduced internal heat production. Previous studies by Kuusela et al. [23], Golden et al. [24], and Ansah et al. [25] reported similar temperature drops with dexmedetomidine, propofol, and isoflurane. Although these factors played a role in the overall temperature decrease, no significant differences were found between the groups before anesthesia induction.

## 5. Conclusions

This study found that placing a hot water bottle under the abdomen, along with a cloth blanket covering the entire body, was more effective in maintaining rectal temperature in cats during head MRI examinations compared to using only a cloth blanket.

## Data Availability

The data generated in this study are presented in this article. For any further information, the reader can contact the authors.
